# Anti-*Trichomonas gallinae* activity of essential oils and main compounds from Lamiaceae and Asteraceae plants

**DOI:** 10.3389/fvets.2022.981763

**Published:** 2022-09-09

**Authors:** María Bailén, Irene Díaz-Castellanos, Iris Azami-Conesa, Sara Alonso Fernández, Rafael A. Martínez-Díaz, Juliana Navarro-Rocha, María Teresa Gómez-Muñoz, Azucena González-Coloma

**Affiliations:** ^1^Department of Preventive Medicine, Public Health and Microbiology, Faculty of Medicine, Autonomous University of Madrid, Madrid, Spain; ^2^Department of Animal Health, Faculty of Veterinary Sciences, University Complutense of Madrid, Madrid, Spain; ^3^Centro de Investigación y Tecnología Agroalimentaria de Aragón, Unidad de Recursos Forestales, Zaragoza, Spain; ^4^Instituto de Ciencias Agrarias, Centro Superior de Investigaciones Científicas (CSIC), Madrid, Spain

**Keywords:** natural products, *Salvia*, *Satureja montana*, *Lavandula luisieri*, linalyl acetate, thymol, 4-terpineol, γ-terpinene

## Abstract

*Trichomonas gallinae* is a flagellated protozoan that parasitizes the upper digestive tract of various bird species and causes avian trichomonosis. The emergence of resistant strains to the standard treatment, based on nitroimidazoles, increases the need to find alternative therapies. In this study, 36 essential oils (EOs) from Lamiaceae and Asteraceae plant families were tested against *T. gallinae* trophozoites using the 3-(4,5-dimethylthiazol-2-yl-)-2,5-dipheniltetrazolium bromide (MTT) reduction assay. Among them, EOs from distinct species of Lamiaceae, including the genera *Lavandula, Salvia, Thymus, Origanum*, and *Satureja* were the ones reporting better anti-trichomonal activity, and were selected for further analysis, including chemical composition and *in vitro* assays. The chemical composition of the selected EOs was determined by gas chromatography followed by mass spectrometry and 19 pure compounds were tested against the protozoa, according to their higher abundance in the active EOs. Pure compounds which displayed the highest activity against *T. gallinae* trophozoites, ordered by highest to lowest activity, were α and β-thujones, camphene, β-pinene, linalyl acetate, thymol, 4-terpineol, γ-terpinene, α-pinene, p-cymene, D-fenchone and β-caryophyllene. A dose dependent effect was observed in most of the EOs and pure compounds tested. The toxicity test conducted in eukaryotic cell cultures with the anti-trichomonal active pure compounds showed that β-caryophyllene, camphene, α-pinene, and β-pinene were slightly toxic for Vero cells, and the selectivity index was calculated. Based on the anti-trichomonal activity and the absence of cytotoxicity results, natural products from Lamiaceae plants could be useful as alternative therapy against avian trichomonosis, mainly those containing linalyl acetate, thymol, 4-terpinenol, γ-terpinene, p-cymene and D-fenchone.

## Introduction

*Trichomonas gallinae* (*T. gallinae*) is a flagellated parasite of the oropharynx and causes a potentially life-threatening disease, oral trichomonosis. The main reservoirs are Columbiformes, but many avian species, including domestic and wild birds, can be infected (1). Nitroimidazoles are the drugs of choice for treatment, but several studies reported resistant strains, both, *in vivo* (2–5) and *in vitro* (3, 4, 6–8). Since the ban of the use of nitroimidazoles for prevention in the EU and the USA, there is no alternative to prevent the disease (9).

Natural products are compounds obtained from living organisms that contain secondary metabolites that help them to survive in adverse environments or even to defend against pathogens. Among them, Essential Oils (EOs) are one of the more frequently employed for diverse uses, including antioxidant, anti-inflammatory and antimicrobial activity, which encompass virus, bacteria, fungi, and parasites (10, 11). Their composition is variable depending on the species, soil conditions, fertilizers, origin, climate, and mode of extraction, as examples, and for that reason it is difficult to assess their effects. Between 10 and 60 components are usually found in their composition, although up to 200–400 different substances are described in some of them (10, 11). However, only the major components, usually 2–4, are thought to be responsible for the attributed properties. Terpenes, especially monoterpenes and sesquiterpenes, are considered the most important components of EOs.

There is little information on the use of natural products against *T. gallinae* and few reports were found in the literature, including some EOs from the Asteraceae and Geraniaceae families (12, 13) but also from other medicinal plants such as *Cymbopogon flexuosus* (14) and *Dennetia tripetala* (15). Besides EOs, aqueous, alkaloid and ethanol and methanol extracts from different plants have been tested against *T. gallinae*, including plants from Lamiaceae and Asteraceae families, such as *Lavandula angustifolia, Rosmarinus officinalis* and *Artemisia annua*, and also extracts from other plants: *Allium sativum, Harungana madagascariensis, Zingiber officinale, Myrtus commuis, Zataria multiflora, Quercus persica, Lycopus europaeus, Pulycaria disenterica, Eugenia uniflora, Murraya koenigii, Peganum harmala, Clausena lansium* (15–24). However, while EOs displayed activity against *T. gallinae*, most of the above cited extracts were less active, and only four publications mention anti-trichomonal (AT) activity of alcoholic or alkaloid extracts comparable to that of metronidazole at a similar dose, extracts from *Clausena lansium* (18), *Peganum harmala* (19), *Eugenia uniflora* (20), *Lavandula angustifolia*, and *Zingiber officinale* (24).

The research of natural products against *Trichomonas vaginalis* (*T. vaginalis*) is a little wider, and EOs from several plant genera, such as *Lavandula, Nectandra* and *Nigella* were tested with satisfactory anti-trichomonal effects, and around 30 compounds from potatoes, tomatoes, and several medicinal plants have been identified having anti-protozoal activities in different bioassays [revised in Friedman et al. (25)].

EOs components from the families Lamiaceae and Asteraceae have shown antimicrobial and insect repellent properties (11), but only a scarce number of publications proved their activity against trichomonads. In this study, we have analyzed the potential use as anti-*T. gallinae* of 36 EOs obtained by two classical methods of EOs extraction: hydrodistillation (HD) and steam distillation (SD). We have also evaluated the main compounds from these EOs against the avian parasite *Trichomonas gallinae* and their cytotoxic effects on African green monkey kidney cells (Vero cells).

## Materials and methods

### Plants and essential oil extraction

The EOs employed in this study were obtained from 17 species from the Lamiaceae plant family and three species from the Asteraceae plant family selected for their medicinal properties, mainly antimicrobial properties. The plant species were cultivated in distinct locations of Spain. *Ditrichia graveolens* in Castilla-La Mancha, and the rest of plants in Aragon: *Santolina chamaecyparissus, Lavandula lanata, Lavandula angustifolia, Lavandula x intermedia “*Abrial”*, Lavandula x intermedia* “Super”, *Lavandula mallete, Origanum majorana, Rosmarinus officinalis, Satureja montana, Mentha suaveolens, Salvia officinalis, Salvia hibrida, Salvia sclarea, Thymus vulgaris, Thymus zygis, Origanum virens*, and *Lavandula luisieri* populations “1” and “2”, this last two populations were predomesticated from two origins, west and center of the Iberian Peninsula. The seeds from the 17 species were deposited in the germplasm of CITA (Centro de Investigación y Tecnología Agroalimentaria de Aragón, Unidad de Recursos Forestales, Zaragoza, Spain).

The plant species used were identified by Dr. Daniel Gomez, IPE-CSIC (see Voucher numbers at Supplementary Table S1). The *Salvia* hybrid (*S. officinalis* L. × *S. lavandulifolia* Vahl) used in this work was obtained by J. Burillo (CITA) (Supplementary Table S1). Aerial plant parts were collected at the flowering stage during the years 2016–2019. Approximately 100 g of aerial parts, maintained at room temperature and preserved from the light at the laboratory for 7 days, were submitted to hydrodistillation. The hydrodistillation was carried out in triplicate with 100 g of dried aerial plant parts and 2 l of water for 2 h in a Clevenger-type apparatus according to the method recommended by the European Pharmacopoeia (26). The oils were dried over MgSO4, filtered and stored at 4°C until used in 2021 for activity and determination of EOs composition, as previously described. (27). Pilot plant steam distillation was carried out on the fresh biomass of the plants (60 Kg total fresh plant biomass) harvested at the flowering stage. A stainless-steel pilot extraction plant equipped with a pressure reducing valve was used as described (28). The pressure of work was 0.5 bar. The hydrolate (aqueous phase) was decanted from the essential oil collected in the condensation section and filtered.

### EOs analysis and pure compounds identification

The EOs from the studied plants were analyzed and the main compounds determined. The analysis was performed by gas chromatography-mass spectrometry (GC-MS) using a Shimadzu GC-2010 Plus coupled to a Shimadzu GCMS-QP2010-Ultra mass detector with an electron impact ionization source at 70 eV and a Single Quadrupole analyzer and employing Helium as carrier gas. Chromatography was carried out with a Teknokroma TRB-5 (95%) Dimethyl- (5%) diphenylpolysiloxane capillary column, 30 m x 0.25 mm ID and 0.25 μm phase thickness. The working conditions used were: Split mode injection using 1 μl of sample with a split ratio (20:1) employing a Shimadzu AOC-20i automatic injector, injector temperature 300°C, transfer line temperature connected to the mass spectrometer 250°C, and ionization source temperature 220°C. The initial column temperature was 70°C, heating up to 290°C at 6°C /min and leaving at 290°C for 15 min. All the samples (4g/μl) were previously dissolved in 100% dichloromethane (DCM) for injection.

The mass spectra, retention time, and retention indexes were used to identify the compounds by comparison with those found in the Wiley database (Wiley 275 Mass Spectra Database, 2001) and NIST 17 (NIST/EPA/NIH Mass Spectral Library), while the relative area percentages of all peaks obtained in the chromatograms were used for quantification. Identification of trans-α-necrodyl acetate from *L. luisieri* was preformed using a standard compound previously isolated.

### Pure compounds

After analyzing the composition of the EOs by GC-MS, products that fulfill the following criteria were selected to study their antitrichomonal activity *in vitro*: three more abundant in each of the selected active EO, abundance higher than 5%, and availability. Some of the major compounds that were excluded were not identified, not commercially available or not easy to obtain or isolate. Pure compounds (monoterpenes and sesquiterpenes) were obtained from commercial sources, except camphor that was previously isolated in our laboratory (ICA, CSIC). Linalool, thymol, α-pinene, α-terpineol, β-pinene, camphene, β- caryophyllene and caryophyllene oxide were obtained from Sigma Aldrich (Madrid, Spain); linalyl acetate, γ-terpinene, and p-cymene from Acros Organics (Madrid, Spain); 4-terpineol from Merck Life Sciences (Madrid, Spain); α and β thujone from Phytolab: borneol, 1,8-cineole, carvacrol and D-fenchone from Fluka (Madrid, Spain).

### Anti-trichomonal activity *in vitro*

The AT activity assay was performed using round bottom microwell plates in quadruplicate. Each well had 150 μl of *T. gallinae* trophozoites in Trypticase-Yeast Extract-Maltose medium (TYM) with 10% fetal calf serum (Sigma, Madrid, Spain) at 500.000 trophozoites/ml (29). EOs and pure compounds were solved in DMSO (Sigma, Madrid, Spain) (<1% final concentration). The EOs were tested under concentrations of 800, 400, and 200 μg/ml, and in those cases that showed moderate antitrichomonal activity (>50%) at the lowest concentration, the assay was done also at lower concentrations: 100, 50, and 25 μg/ml. Trophozoite viability was analyzed using the modified MTT colorimetric assay method as previously described in literature (29). Briefly, after incubating the plate for 24 h at 37°C with the EOs and compounds to be tested, the plates were centrifuged at 750 x *g*, the medium was eliminated and 100 μl of a solution of MTT (Sigma, Madrid, Spain) (1.25 μg/ml) and PMS (Sigma, Madrid, Spain) (0.1 μg/ml) in PBS (Sigma, Madrid, Spain) was added to each well. The plate was incubated for 45 min in dark conditions to allow the reduction of MTT to formazan salts. Prior to reading, 100 μl of DMSO was added to each well to dissolve the formazan crystals. The plate was read in a spectrophotometer using a wavelength of 570 nm.

The percentage of AT activity was calculated as growth inhibition using the following formula:


$$\begin{array}{l} \%\;AT\;=\;100-\left[\left(Ap-Ab\right)÷\left(Ac-Ab\right)\right]\times 100 \end{array}$$

Where Ap is the absorbance of the tested product, Ab the absorbance of the blank and Ac the absorbance of the control wells (culture without treatment).

Pure compounds were assayed using the same method described above, although the concentrations tested were 100, 75, 50, 25, 10, and 1 μg/ml. Metronidazole (Acros Organics, Madrid Spain) was used as a reference drug for antitrichomonal activity.

### Cytotoxicity of active pure compounds

African green monkey kidney cells (Vero cells) were grown in Dulbecco's modified Eagle's minimal essential medium (DMEM, Avantor, Llinars del Vallès, Barcelona) supplemented with 10% fetal calf serum (Sigma, Madrid, Spain) and 1% penicillin/streptomycin (Fisher Scientific, Madrid, Spain) at 37°C under a humidified atmosphere of 5% CO_2_/95% air.

Cells seeded in 96-well flat-bottom microplates with 100 μL medium per well (initial densities 10^4^ cells per well) were exposed for 48 h to serial dilutions of the test compounds in DMSO (<1% final concentration). Cell viability was analyzed by the MTT colorimetric assay method, and the purple-colored formazan precipitate was dissolved with 100 μL of DMSO (30). The cell viability was tested with each compound in a dose-response experiment to calculate their relative potency (CC_50_) value, the effective dose to give 50% cell viability, employing the concentrations of 100, 75, 50, 25, 10, and 1 μg/ml.

IC_50_ (μg/ml) reflects the dose needed to produce 50% mortality of *T. gallinae* trophozoites. Selectivity index was calculated for the AT active products that showed cytotoxicity, using the formula SI = CC_50_/IC_50_. Compounds with SI higher than 1 were considered as potential antitrichomonal compounds since they are more toxic for trichomonads than for mammalian cells.

### Statistical analysis

The data was analyzed using STATGRAPHICS Centurion XIX (https://www.statgraphics.com).

IC_50_ anti-trichomonal activity and CC_50_ cytotoxic activity were determined from the dose-response experiment, employing a linear regression analysis (% cell viability on log dose).

Parametric bivariate correlation analysis was performed between the main components of the EOs and the AT activity. The chemical composition of the 36 EOs studied was analyzed and those components in proportion higher than 5% were correlated with the IC_50_ of the EOs.

## Results

### Anti-trichomonal activity of EOs from Lamiaceae and Asteraceae plants

The activity against *T. gallinae* trophozoites of a total of 36 EOs belonging to 17 different plant species was evaluated in this study, including species of the Lamiaceae genera *Lavandula, Origanum, Salvia, Satureja, Mentha*, and *Thymus*, and the Asteraceae genera *Ditrichia* and *Santolina* (Table 1). From the tested EOs, 17 were extracted by steam distillation (SD), while the remaining 19 were obtained by hydrodistillation (HD).

**Table 1:** Effects of the tested essential oils on *Trichomonas gallinae* (IC_50_)^a^.

**Family**	**Genus**	**Species**	**Extraction method**	**IC_50_ (μg/ml)**
Asteraceae	*Santolina*	*Santolina chamaecyparissus*	HD	394.3 (316.8–490.7)
	*Ditrichia*	*Ditrichia graveolens*	SD	261.0 (203.7–334.5)
		*Ditrichia graveolens*	HD	259.7 (233.9–288.2)
Lamiaceae	*Lavandula*	*Lavandula lanata*	HD	731.7 (546.1–980.2)
		*Lavandula luisieri* 1	SD	189.8 (170.0–212.0)
		*Lavandula luisieri* 1	HD	331.4 (278.8–394.0)
		*Lavandula luisieri* 2	SD	103.4 (77.7–137.7)
		*Lavandula luisieri* 2	HD	455.2 (434.5–476.8)
		*Lavandula angustifolia*	SD	600.4 (480.6–749.9)
		*Lavandula angustifolia*	HD	823.4 (612.1–1.107.8)
		*Lavandula x intermedia* “Abrial”	SD	406.8 (286.3–578.0)
		*Lavandula x intermedia* “Abrial”	HD	452.7 (370.9–552.6)
		*Lavandula x intermedia* “Super”	SD	583.7 (445.8–764.2)
		*Lavandula x intermedia* “Super”	HD	321.2 (263.4–391.6)
		*Lavandula mallete*	SD	702.1 (558.3–883.0)
		*Lavandula mallete*	HD	373.9 (318.4–439.1)
	*Origanum*	*Origanum virens*	SD	175.4 (123.4–249.3)
		*Origanum virens*	HD	197.9 (106.2–368.8)
		*Origanum majorana*	SD	139.7 (109.1–178.9)
		*Origanum majorana*	HD	158.8 (129.9–194.0)
	*Rosmarinus*	*Rosmarinus officinalis*	SD	328.1 (292.8–367.8)
		*Rosmarinus officinalis*	HD	256.6 (227.6–289.4)
	*Satureja*	*Satureja montana*	SD	141.4 (128.9–155.0)
		*Satureja montana*	HD	321.3 (244.4–422.6)
	*Mentha*	*Mentha suaveolens*	SD	419.1 (355.5–494.0)
		*Mentha suaveolnes*	HD	303.0 (262.8–349.3)
	*Salvia*	*Salvia officinalis*	SD	139.6 (119.1–163.6)
		*Salvia officinalis*	HD	420.4 (396.4–445.9)
		*Salvia hibrida*	SD	134.6 (118.1–153.4)
		*Salvia hibrida*	HD	239.9 (196.9–292.3)
		*Salvia sclarea*	SD	712.3 (606.1–837.1)
		*Salvia sclarea*	HD	117.4 (99.4–138.6)
	*Thymus*	*Thymus vulgaris*	SD	193.3 (161.9–230.9)
		*Thymus vulgaris*	HD	166.0 (151.1–182.4)
		*Thymus zygis*	SD	274.5 (237.2–317.6)
		*Thymus zygis*	HD	133.1 (117.5–150.7)

^a^IC50 (μg/ml) = concentration needed to produce 50% trophozoite mortality. SD, steam distillation; HD, hydrodistillation.

**Figure 1:**
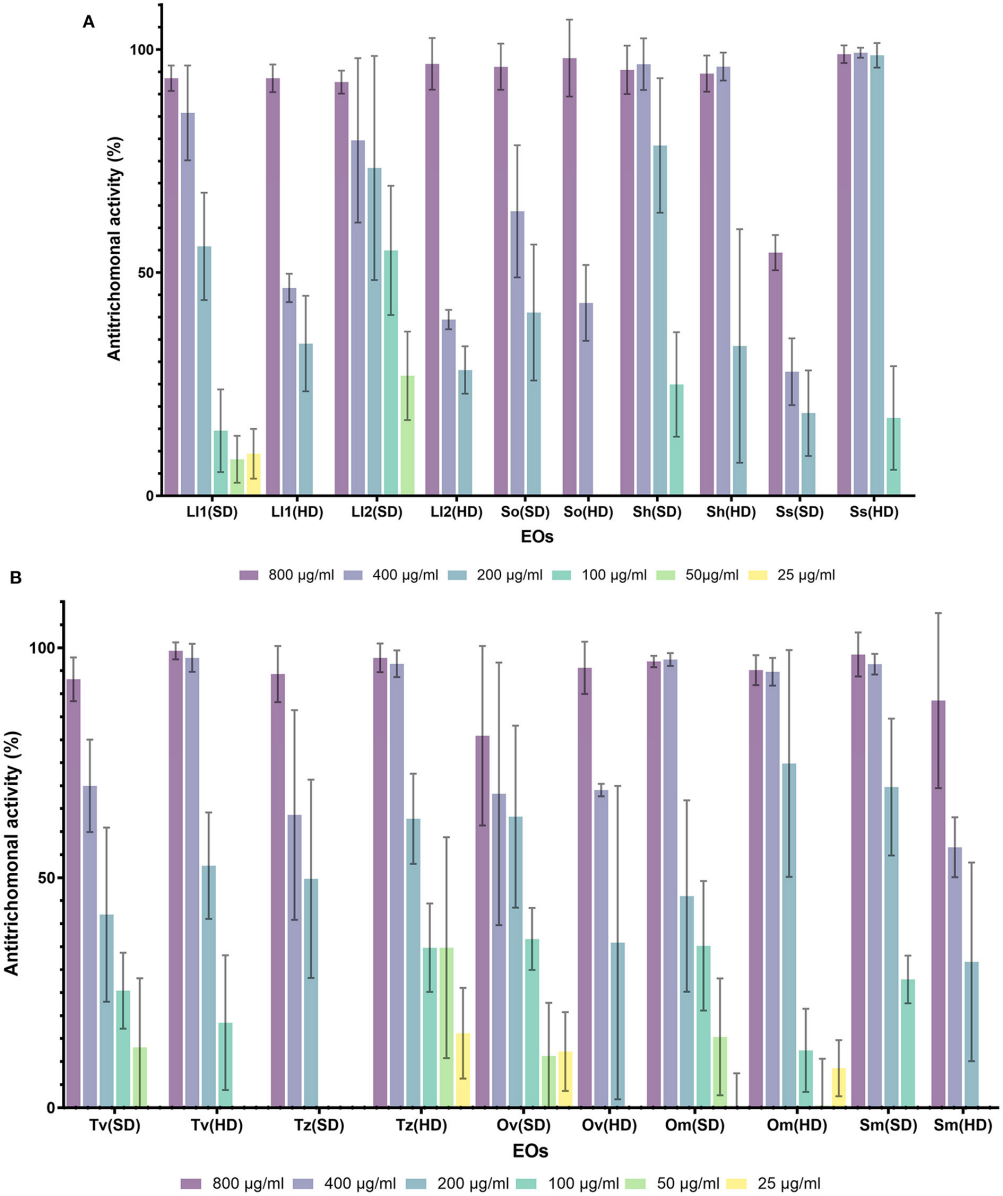
Percentage of antitrichomonal activity of EOs obtained by hydrodistillation (HD) and steam distillation (SD). **(A)** EOs from *L. luisieri* 1 and 2 (Ll1 and Ll2), *S. officinalis* (So), *S. hibrida* (Sh) and *S. sclarea* (Ss). **(B)** EOs from *T. zygis* (Tz), *T. vulgaris* (Tv), *O. virens* (Ov), *O. majorana* (Om) and *S. montana* (Sm).

In general, all the tested EOs, except two (EOs from *L. lanata* and *L. angustifolia*), displayed good anti-trichomonal activity (higher than 80%), especially at the highest concentration (800 μg/ml), although the results vary depending on the plant and the extraction method used in each case (Figure 1; Supplementary Figure 1).

A total of 13 EOs from five species of *Lavandula* were analyzed (Figure 1; Supplementary Figure 1), of which seven EOs from three species showed high activity (80–100%) at the highest concentration. These belong to the species *L. luisieri, L. x intermedia* “Abrial” and “Super” and *L. mallete*. It is remarkable that *L. luisieri* was the only one that showed high activity in all the tested EOs coming from two populations and two extraction methods, although in both populations the best results were reported by the EOs extracted by SD. EOs from *L. luisieri* presented moderate activity (above 50%) even at lower concentrations, 200 μg/ml in the case of population 1, and 100 μg/ml in population 2, being the EOs with the highest activity among the *Lavandula* species.

All the tested EOs from *Salvia* species showed activity above 90% at the highest dose (800 μg/ml) except the EO from *S. sclarea* obtained by SD, which displayed 54.5% AT activity (Figure 1). By contrast, the EO obtained by HD from the same species, *S. sclarea*, maintained a high activity, close to 100%, even at lower concentration (200 μg/ml). On the contrary, the EOs of *S. officinalis* and *S. hibrida* obtained by SD displayed better AT activity than EOs obtained by HD, keeping moderate activity even at 200 μg/ml.

All the EOs from *Thymus* spp. and *Origanum* spp. (Figure 1) presented AT activity over 80% at the highest concentration (800 μg/ml). The activity was maintained at intermediate concentrations (400 μg/ml) with EOs from *T. vulgaris* and *T. zygis* obtained by HD, as well as for EOs from *O. majorana* obtained by both methods. It is noteworthy that the EO from this last species obtained by HD kept AT activity over 70% at 200 μg/ml.

In the same manner, the EOs from the other Lamiaceae tested, *R. officinalis, S. montana* and *M. suaveolens*, (Figure 1; Supplementary Figure S1) showed high activity (80–100%) at the highest concentration employed (800 μg/ml), except for *M. suaveolnes* obtained by SD, whose AT activity was close to 70%. The best result was obtained with EO from *S. montana* extracted by SD, which maintained an activity close to 70% at 200 μg/ml.

Finally, all the EOs from Asteraceae tested in this study (Supplementary Figure S1) showed AT activity over 90% at the highest concentration tested (800 μg/ml). This activity downed close to 60% at 400 μg/ml in both samples of *D. graveolens* and declined drastically in the EO from *S. chamaecyparissus*.

The IC_50_ from the tested EOs was calculated (Table 1), and those having a IC_50_ lower than 200 μg/ml were selected for further analysis (*n* = 13), together with the EOs obtained from the alternative extraction method to compare their composition. Among all the plants, those with EOs showing the highest AT activity were *L. luisieri* (2 populations), *S. officinalis, S. hibrida, S. sclarea, T. vulgaris, T. zygis, O. majorana*, and *S. montana*.

From the 13 EOs, eight were obtained by SD while five were obtained from HD. Among the selected species, most of them rendered better results with EOs obtained by SD than EOs obtained from HD (*L. luisieri* 1 and 2, *O. majorana, O. virens, S. hibrida, S. officinalis*, and *S. montana*), and only three of them display better results with EOs obtained by HD (*S. sclarea, T. vulgaris* and *T. zygis*), although in some cases differences in the IC_50_ were scarce.

### Composition of essential oils

The composition of the EOs with the highest AT activity was determined by GC-MS (Table 2) and compared with the composition of the EO extracted by the alternative method (SD or HD). In some of the active EOs, more than fifty compounds were identified, but only the major components (with a proportion higher than 5%) were considered.

**Table 2:** Chemical composition of the EOs from the most active species*: L. luisieri* (1 and 2) (Ll1 and Ll2), *S. montana* (Sjm)*; S. officinalis* (So)*, S. hibrida* (Sh)*, S. sclarea* (Ss)*, T. vulgaris* (Tv)*, T. zygis* (Tz)*, O. majorana* (Om) and *O. virens* (Ov). RI, Retention index; RT, Retention time; HD, EOs obtained by hydrodistillation; SD, EOs obtained by steam distillation.

**Compounds**	**RI**	**RT**	**Ll 1**		**Ll 2**		**Sjm**		**Sh**		**So**		**Ss**		**Tv**		**Tz**		**Om**		**Ov**	
			**HD**	**SD**	**HD**	**SD**	**HD**	**SD**	**HD**	**SD**	**HD**	**SD**	**HD**	**SD**	**HD**	**SD**	**HD**	**SD**	**HD**	**SD**	**HD**	**SD**
α-pinene	937	3.81	5.7	4.9	2.8	2.6	0.9	0.4	4.3	6.3	3.4	4.4	0.1	0.3	1.1	1.4	1.7	0.4	0.8	0.5	0.7	0.4
Camphene	950	4.02	0.6	1.1			0.5	0.2	6.6	7.6	2.8	3.5	0.6	0.4	0.5	1.1	2.1	0.5	0.1	0.4	0.3	0.2
Sabinene	974	4.34							0.7	1.4	0.2	0.5							5.8		2.7	1.1
β-pinene	982	4.40	0.2				0.4	0.2	10.9	18.4	7.5	12.0	0.1	1.5	0.5	0.5	0.4	0.2	0.6	0.3	0.5	0.3
β-myrcene	988	4.53					1.4	1.1	4.1	4.2	0.6	1.9	2.0	0.3	1.6	0.7	1.5	0.8	1.3	0.8	6.0	2.1
α-terpinene	1,020	5.00					2.6	1.9	0.1	0.3	0.1	0.3			1.4	1.0	1.6	0.5	5.9	1.0	3.1	2.3
p-cymene	1,026	5.15	0.7	0.6			18.5	11.8	2.4	1.6	0.7	0.9		0.7	22.3	31.7	18.3	8.0	5.5	21.0	12.2	3.3
1.8-cineole	1,034	5.24	4.5	1.1	0.9	2.0	0.9	0.9	20.6	13.4	18.0	12.8		0.7	2.3	2.3		1.5	3.4	2.1		1.2
Ocimene	1,043	5.48		0.5					0.1	0.2		0.1	0.4					0.4	5.4		2.7	1.9
γ-terpinene	1,060	5.72					11.7	12.4	0.4	2.3	0.2	1.3			5.7	2.8	9.0	1.9	10.7	1.2	22.4	15.4
D-fenchone	1,089	6.31	20.0	1.3	2.5	1.3																
Linalool	1,102	6.45	4.2	0.3	1.1	2.2	1.3	1.1	0.4	0.1	0.5	0.2	22.7	2.0	3.8	3.5	5.4	11.6	3.0	3.5	13.9	15.3
β-thujone	1,109	6.64							3.2	2.0	22.9	13.9		0.3								
α-thujone	1,143	6.86							0.6	0.4	6.0	4.2										
Camphor	1,148	7.44	13.4	35.0	49.2	4.6	0.1	0.1	14.2	6.4	5.0	4.7	0.2	1.2	0.5	0.9	1.0	2.7		1.0	0.1	2.4
Borneol	1,160	7.80			1.3	1.6	2.0	1.3						2.4	1.0	2.0	6.3	2.5		0.9	0.7	1.6
Trans-bornyl acetate	1,165	7.88							12.9	6.9	7.0	3.9		1.1					0.1			
4-terpinenol	1,181	8.19	0.7				1.5	0.9	0.7	0.2	0.5	0.2			1.5	0.8	0.8	1.4	29.7	0.9	2.6	1.5
α-terpineol	1,196	8.40					0.2	0.2			0.2		11.3	0.4	0.3	0.2	0.1	0.6	3.6	0.2	0.3	0.7
Carvacryl methyl ether	1,240	9.54																		3.5	5.7	2.3
Linalyl acetate	1,257	9.70											31.2	14.5		0.1		17.9			0.2	15.1
123/139/81/121/79/91/57/105/43/124*	1,282	10.47				5.6																
Trans-α-necrodyl acetate	1,283	10.47	12.4	18.8	13.2	18.1																
Lavandulyl acetate	1,285	10.53	5.6	6.2																		2.6
Lavandulol	1,286	10.54			5.9	7.9																
Thymol	1,287	10.58					17.3	7.1							43.8	28.2	39.5	21.2		32.7	6.6	4.6
Carvacrol	1,299	10.84					32.6	41.3							2.4	2.3	5.0	1.4		2.2	4.7	2.9
Neryl acetate	1,360	12.21											7.5	0.4			0.7					0.6
β-caryophyllene	1,421	13.55	0.4	0.6	0.1	3.7	2.1	6.1	3.9	8.0	6.4	10.3	3.0	12.2	2.4	5.8	1.4	4.0	5.2	7.3	2.1	4.3
α-humulene	1,458	14.29		0.5			0.1	0.3	1.0	1.7	4.3	5.6	0.2	2.9	0.1	0.3			0.6	0.8	0.2	
Germacrene D	1,484	14.87	0.4	0.6		7.5		0.3		0.1	0.1	0.1	6.0	3.5	0.1	0.1		1.3	2.5	0.3	0.4	2.4
Spatulenol	1,580	16.90				1.5		0.2	1.7	1.3		0.8	0.2	5.4			0.2		0.6	0.2	1.7	0.2
Caryophyllene oxide	1,587	17.02	0.4		0.1	1.2	1.0	1.4	1.1	1.0	0.9	0.7	0.6	6.5	1.1	1.7	0.9	0.8	1.5	3.7	1.1	0.3
Viridiflorol	1,594	17.20	1.7	1.4		2.1			3.8	4.9	7.4	7.8		15.0						0.5		
α -bisabolol	1,684	18.94																5.5				2.2
Epimanool	2,059	25.63		0.8					0.3	0.7	1.4	1.8	0.3	7.4						1.1		

*Mass spectra of a nonidentified compound.

% Area: 
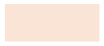
 5–10% 
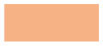
 10–15% 
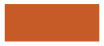
 >15%

Camphor, trans-α-necrodyl acetate and D-fenchone were the main compounds of *L. luisieri* EOs (both populations), with concentrations over 15% in any of the populations tested (Table 2). The extraction method influenced the composition of the oils, with D-fenchone being the major component in the EO of population “2” obtained by HD and trans-α-necrodyl acetate in both EOs obtained by SD. Camphor was more variable with the extraction method, and appear at higher concentration in the EO from *L. luisieri* “1” obtained by SD and *L. luisieri* “2” obtained by HD.

The main composition of the EOs of the three species of *Salvia* analyzed shows a large list of compounds (Table 2). Differences were observed between the species, as well as considering the extraction methods to obtain the EOs. The EOs of *S. officinalis* present β-pinene, 1,8-cineole, β-thujone and β-caryophyllene as the major components, representing over 10% in the composition depending on the extraction method. β-caryophyllene and β-pinene were the major components in the SD extract, while β-pinene and 1,8-cineole were more abundant in the EO obtained by HD. Among the major compounds of the EOs from *S. hibrida*, 1,8-cineole, camphor, and borneol were present over 10%, mainly in the HD extract, while β-pinene camphene and β-caryophyllene were more abundant in the EOs extracted by SD. The EO from *S. sclarea* was the most different in composition compared to the other two species. Linalyl acetate stands out in the composition of both extracts, although they differ in the proportion, being more than double in the HD one. Linalool and α-terpineol were only majority in the HD extract and β-caryophyllene and viridiflorol in the SD oil.

Among the major components in the extracts of the genus *Thymus* (Table 2), p-cymene and thymol appeared in high proportions in the EOs of *T. vulgaris* and *T. zygis*, while linalool and linalyl acetate appeared in high proportions (over 10%) in the EO from *T. zygis* obtained by SD. The concentration of p-cymene varies among the extracts, among the species and among the extraction method, being higher in the EO of *T. vulgaris* obtained by SD and in the EO from *T. zygis* by HD. The concentration of thymol was over 20% in all the EOs, but the percentage varies according to the extraction method, appearing in higher concentration in the EO obtained by HD.

The composition of *Origanum* EOs (Table 2) varied depending on the species (*O. virens* and *O. majorana*) and the extraction technique used. In the EO obtained by SD, the major components were p-cymene and thymol, with concentrations over 20%. Thymol was the major component of the EO obtained by SD, with an abundance of 32.73%, while in the HD extract it did not even appear. On the other hand, γ-terpinene and 4-terpineol displayed an abundance over 10% in the EO from *O. majorana* obtained by HD. In the EO from *O. virens* obtained by SD, only linalyl acetate appeared at a concentration higher than 15%, while p-cymene, γ-terpinene, and linalool appeared at concentrations higher than 10% in the EO obtained by HD.

The main compound of *S. montana* EOs was carvacrol, representing over 30% in the EOs obtained from the two extraction methods. Other compounds with concentrations higher than 10% in any of the EOs were γ-terpinene, p-cymene and thymol (Table 2).

According to the extraction method and comparing the composition of the EOs obtained in the distinct species analyzed, EOs obtained by SD in comparison with EOs obtained by HD, showed systematically higher proportion of the following compounds: β-pinene, β-caryophyllene, viridiflorol, and trans-α-necrodyl acetate. The opposite was observed with the compounds D-fenchone, camphene, α-terpineol, α-terpinene, 4-terpineol, that were extracted in higher proportion with the HD method compared to the SD method. The rest of the compounds appeared with higher enrichment in one or another of the extraction methods used, but not systematically (i.e., thymol, p-cymene, camphor, linalool, linalyl acetate, γ-terpinene, α-pinene).

Parametric bivariate correlation analysis between the main components of the EOs and the AT activity showed seven significant correlations with AT (*p* < 0.05) (Table 3). Among the compounds included in the analysis, p-cymene, thymol, γ-terpinene, and α-terpinene were significantly associated with the AT activity of the EOs (negative correlation between compound abundance and IC_50_ of the EO). On the contrary, 1,8-cineole, linalool and lavandulol were significantly associated (*p* < 0.05) with lower AT activity (positive correlation between compound abundance and IC_50_ of the EO).

**Table 3:** Pearson correlation coefficients between main components of EOs and AT activity.

**Compound**	**Pearson correlation coefficient**	**p**
α-terpinene	−0.374	0.025
p-cymene	−0.385	0.020
1,8-cineole	0.413	0.012
γ-terpinene	−0.373	0.025
Linalool	0.334	0.047
Lavandulol	0.335	0.046
Thymol	−0.355	0.033

### Antitrichomonal activity and cytotoxic activity of pure compounds

A total of 19 pure compounds, 17 monoterpenes and two sesquiterpenes, were tested against *T. gallinae* (Supplementary Figure S2). All of them were major components of the active EOs and fulfill the criteria previously described.

Among the tested products, seven did not showed AT activity at 100 μg/ml, including borneol, camphor, carvacrol, 1,8-cineole, caryophyllene oxide, linalool and α-terpineol. The rest of the tested compounds showed AT activity, and the IC_50_ was calculated. From higher to lower AT activity, these compounds were: α and β thujones, camphene, β-pinene, linalyl acetate, thymol, 4-terpinenol, γ-terpinene, α-pinene, p-cymene, D-fenchone, and β-caryophyllene (Table 4).

**Table 4:** Effects of the tested compounds on *Trichomonas gallinae* and Vero cells (IC_50_ and CC_50_ respectively).

**Compound**	**Vero cells (CC_50_^a^)**	***T. gallinae* (IC_50_^b^)**	**SI^c^**
1,8 cineole	>100	>100	1.0
Linalool	>100	>100	1.0
Thymol	>100	38.4 (31.5–46.9)	2.6
Carvacrol	>100	>100	1.0
Linalyl acetate	>100	32.2 (27.2–38.2)	3.1
γ-terpinene	>100	43.8 (37.0–52.0)	2.3
p-cymene	>100	59.7 (49.3–72.4)	1.7
α-terpineol	>100	>100	1.0
Caryophyllene oxide	>100	≈100	1.0
α-pinene	88.9 (88.0–89.7)	44.2 (42.3–46.1)	2.0
β-pinene	88.2 (87.2–89.2)	29.6 (26.2–33.4)	3.0
Camphor	>100	>100	1.0
Borneol	>100	>100	1.0
D-fenchone	>100	61.9 (54.3–70.6)	1.6
β-caryophyllene	60.8 (56.9–65.0)	86.1 (78.3–94.7)	0.7
α and β thujone	>100	17.3 (11.3–26.3)	5.8
4-terpinenol	>100	41.5 (33.9–50.7)	2.4
Camphene	74.8 (66.3–84.2)	24.0 (21.9–26.2)	3.1
Metronidazole	>100	1.0 (0.8–1.1)	100.0

^a^CC50 (μg/ml), concentration needed to produce 50% Vero cell mortality; ^b^IC50 (μg/ml), concentration needed to produce 50% trophozoite mortality ^c^SI, Selectivity index.

The cytotoxic activity of the 19 pure compounds was also evaluated. None of the products without AT activity displayed cytotoxic activity. From the AT active compounds, only four of them were slightly toxic for Vero cells: α-pinene, β-pinene, camphene and β-caryophyllene (Table 4).

Taking these results into account, an adjustment of the effective dose was calculated to equilibrate AT activity and cytotoxic effect. For that purpose, the selectivity index (SI) was applied (Table 4). The best results were obtained by camphene (SI = 3.12), linalyl acetate (SI = 3.11), thymol (SI = 2.6), 4-terpinenol (SI = 2.41), γ-terpinene (SI = 2.28), p-cymene (SI = 1.68), and D-fenchone (SI = 1.62). β-caryophyllene was more toxic for Vero cells than for *T. gallinae* trophozoites and consequently the SI was lower than 1, excluding it for AT purposes. Besides, α and β-thujones were also excluded because they are potent neurotoxic substances (31), although they did not show cytotoxic effect on Vero cells.

## Discussion

Several anti-trichomonas EOs, extracts and compounds from plants have been tested against trichomonads, mainly against the human pathogen *T. vaginalis*. Among them, the Lamiaceae family with more than 230 genera, usually aromatic, and the Asteraceae family, with worldwide distribution and more than 1,600 genera, stand out (32). Species from *Lavandula* and *Salvia* (24, 33), *Artemisia* (12), and *Thymus* (34) have been more extensively explored with these parasites. In our study, the best results were obtained from *L. luisieri* (both populations) while lower activity was observed with other *Lavandula* species or varieties, such as *L. angustifolia* or *L. x intermedia*, which displayed activity against *Giardia, Hexamita* or *T. vaginalis* in other studies (33). These slight disagreement between the studies could be due to differences in the composition, since it may vary with the diverse conditions of growing, climate or part of the plant collected, but also to the selected methodology employed in the present study.

Also, a remarkable activity was observed with EOs from *Salvia* (*S. sclarea, S. hibrida* and *S. officinalis*) and *Thymus* (*T. vulgaris* and *T. zygis*) species, which agrees with the results obtained from other authors testing AT activity (32, 35). Within the list of our best AT active EOs, *Origanum* and *Satureja* species (*O. vulgare* and *O. majorana* and *S. montana*) are included. There are no other anti-trichomonads assays with these two genera, but some authors demonstrated their activity against other protozoa such as *Leishmania* and *Giardia* (35, 36), as well as with some fungi and bacteria (37, 38).

Satisfactory results were also obtained in our study from *R. officinalis* and *M. suaveolens*, the former plant being mentioned in one study with moderate activity against *T. gallinae* (22) and presenting activity against *Plasmodium falciparum* in another study (35). Although the AT activity was good, they were over the cutoff point value in our study (IC_50_ <200 μg/ml), and for that reason they were not included in later experiments.

Other Asteraceae from the genus *Tanacetum* showed anti-trichomonal effect in previous studies (32), but in our case, only *D. graveolens* displayed moderate results at intermediate doses. Due to the lower AT activity, we did not include any of the Asteraceae EOs in further analysis.

Many natural products assayed against trichomonads from the Lamiaceae are EOs, while compounds from other plants were extracted employing other methods, such as ethanolic, methanolic or alkaloid extraction, as examples. Among the extracts tested against *T. gallinae* trophozoites, many needed high amounts of extract to obtain a good AT activity, such as the aqueous garlic (*Allium sativum*) extract (16), and alcoholic extracts of *Lycopus europaeus* and *Pulicaris dysenterica* (21), *Quercus persica, Artemisia annua, Myrtus commuis, Zataria multiflora, R. officinalis, Allium sativum* (22) and *Harungana madagascariensis* (23). Others showed some kind of toxicity, such as the alkaloid extract of *Murraya koenigii* (17). Finally, some of the previously cited assayed extracts were not fully characterized, the major components were not described, or the potential toxic effects were not tested (22), and for these reasons and the differences in composition, we could not include them in the comparison with the result of the present study.

Regarding AT active EOs, four plants have been tested against *T. gallinae* so far, *Dinettia tripetala* (Annonaceae), *Artemisia sieberi* (Asteraceae), *Pelargonium roseum* (Geraniaceae) and *Cymbopogon flexuosus* (Poaceae) (12–15). Except *C. flexuosus* (14), that did not share components with the Lamiaceae tested, the other plants EOs shared components with the ones evaluated by us and will be discussed further.

Some of the common compounds were not highly active against trichomonads when tested alone in the experiments conducted in the present study. For example, linalool was present in the EO composition of *P. roseum* and *D. tripetala*, two plants with AT activity *in vitro* and *in vivo* (13, 15). Although linalool has been suggested as having activity against *Trichomonas*, under our conditions, linalool was not an active AT compound, and the activity of those EOs could be due to other main components, beta-citronellol, geraniol in *P. roseum* EO (13), 2-phenylnitroethane in *D. tripetala* EO (15), ocimene, camphor, linalyl acetate, borneol, 1-8-cineol, and α-pinene in the ethanolic extracts of *L. angustifolia* (24), or even to synergistic effect between them. Besides, linalool showed a positive correlation with the IC_50_ in our study, indicating that those EOs with higher amounts of linalool had lower AT activity. Besides linalool, other compounds with scarce AT activity in our study, but present in the composition of active AT EOs or extract, are camphor and 1,8-cineol in *A. sieberi* EO (12) or the ethanolic extract of *L. angustifolia* (24). In the same way that linalool, 1,8 cineole had an inverse correlation with AT activity.

Thujones, mainly α and β thujones, are the most active anti-*Trichomonas* compounds in our study. These compounds are present also in the composition of *A. sieberi* EOs (12), which was a suitable candidate to treat *T. gallinae*, according to *in vitro* and *in vivo* studies. Although the authors did not report toxicity in the *in vivo* experiment, caution should be taken with these compounds since they are recognized as neurotoxic (31).

Camphene was one of the most active against *T. gallinae* when tested under our conditions. This compound was present in high amounts (higher than 5%) in the EO from *Salvia hibrida*, one of the most active EO tested in the present study, and the abundance of this compound was higher in the EO extracted by SD (7.6%, the most active of both EOs) than the EO extracted by HD (2.8%, the least active of both EOs). Camphene was also present as a major component of *A. sieberi* EO, which was highly active against *T. gallinae* both, *in vivo* and *in vitro* (12). Camphene was slightly cytotoxic for Vero cells, but it might be useful if adjusting the dose according to the SI, since it was the highest one, excepting the potential toxic thujones.

Two compounds are included in the composition of highly active extracts against trichomonads assayed by other authors and the present study: linalyl acetate and α-pinene. They were between the main components of the AT active ethanolic extract of *L. angustifolia* (24) and EOs from *S. sclarea, O. virens*, and *T. zygis*. Linalyl acetate is a suitable candidate as anti-trichomonal, since the IC_50_ for *T. gallinae* trophozoites was low (32.2), and the SI high (3.1), and no toxicity was found in Vero cells. Besides, in the *S. sclarea* EOs, linalyl acetate appears with a concentration of more than double in the HD extract (31.20% in HD vs. 14.48% in SD), which could explain the higher activity of the EO extracted by HD *vs*. the EO extracted by SD. On the other hand, α-pinene was present in considerable amounts in *S. hibrida* EO extracted by SD (6.3%), which was more active than the *S. hibrida* EO extracted by HD (4.3%). The anti-trypanosomatid capacity of some EOs, including *Leishmania major* and *Trypanosoma brucei*, was also attributed to α-pinene (35, 39).

Regarding β-pinene, it has been proven as an active anti-*T. vaginalis* compound in another study (32). Within the major compounds of the *Salvia* species evaluated, β-pinene were found in higher concentration in the extracts of *S. officinalis* and *S. hibrida* obtained by SD, which may explain the higher activity, compared to HD extracts. We must keep in mind the slight toxicity of the compound, but β-pinene is the third compound with better AT activity, and for that reason we still consider that both, α and β-pinenes, are good candidates as anti-trichomonads pure compounds.

There are three compounds that appeared with high abundance in several of the most active EOs evaluated in our study: p-cymene, γ-terpinene, and thymol, which were positively correlated with the AT activity. These three compounds are more abundant in EOs from *T. vulgaris* and *T. zygis* extracted by HD, which displayed higher activity when compared with the EOs extracted by SD. These compounds are also included among the major components of both species of *Origanum, O. majorana* and *O. vulgaris*. The EO of *O. majorana* extracted by SD, which displayed higher AT activity, had higher abundance of thymol and p-cymene, but lower amount of γ-terpinene. We found a similar situation with EOs from *O. vulgare*, but, in any case, IC_50_ from all these EOs were similar, both, for *O majorana* (IC_50_ = 139.7 for EO by SD *vs*. IC_50_ = 158.8 for EO by HD) and for *O. virens* (IC_50_ = 175.4 for EO by SD *vs*. IC_50_ = 197.9 for EO by HD). The compounds were also present at high concentration in the EOs from S. *montana*, although the proportions were higher in the less active EO (extracted by HD). Maybe the higher concentration of carvacrol and β-caryophyllene in the EO from *S. montana* extracted by SD may act synergistically with the other three compounds, as it has been observed in previous studies (40). Two of these compounds, p-cymene and γ-terpinene have also shown activity against *T. vaginalis* (38, 41), and their combination together with thymol in the most active EOs is frequent, possibly acting in a synergistically manner, at it has been previously proven with other protozoa (40). The activity of these compounds have been demonstrated against other protozoa as well, such as p-cymene against *Plasmodium falciparum* (40) and thymol against *T. brucei* and *T. cruzi* (35). There is a high agreement that these three compounds are good AT compounds considering different studies and authors.

In other studies, caryophyllene oxide and β-caryophyllene were found active against *T. vaginalis* (32). However, under our conditions, they displayed a slight activity against *T. gallinae*, and for that reason they are not included within the list of better anti-*T. gallinae* compounds in the present study. The EO from *S. officinalis* extracted SD showed better AT activity, and higher abundance of β-caryophyllene. Also, the EO from *S. officinalis* extracted by HD had higher abundance of 1,8-cineole, a compound that is inversely correlated with the anti-*T. gallinae* activity in our conditions. Maybe antagonistic interactions could be acting between β-caryophyllene and 1,8-cineole.

According to our correlation analyses, assays employing α-terpinene could also be of interest and should be further studied in future assays. Also, Germacrene D, lavandulol, trans-α-necrodyl acetate, and an unidentified compound were present at percentages higher than 5% in the most active EOs tested in the present study, *L. luisieri* “2” extracted by SD. Lavandulol showed a negative correlation with AT activity and therefore discarded for future studies. Trans-α-necrodyl acetate is another interesting compound to evaluate due to its highly abundance (18%) in the most active EO (*L. luisieri* “2” by SD). However, it was not significatively correlated with the IC_50_ of the EOs, which could be due to absence of this compound in other plants EOs.

Comparing the chemical structures of the tested terpenes it is evident that oxidation of the double bound Δ^4, 5^ of β-caryophyllene to produce caryophyllene oxide reduced both cytotoxic and AT effects. For monoterpenes, the double methylation of C7 in compounds with a bicycle-heptane structure decreases the AT activity, whereas the acetylation of the hydroxyl group in C3 for linalool increases the AT effects. Also, the position of the hydroxyl groups in the monoterpene structure affects the AT activity as can be seen by the variations between the activity of 4-terpineol and α- terpineol or between carvacrol and thymol.

Although there were a larger number of highly active EOs extracted by SD (*n* = 9/13) than by HD, it is difficult to recommend one or another method of extraction, since some of the better AT compounds were enriched by SD or HD in a non-consistent manner (p-cymene, linalyl acetate and γ-terpinene), while trans-α-necrodyl acetate, and β pinene were always extracted in higher proportions in EOs obtained by SD and camphene in EOs obtained by HD. Thymol was extracted in higher amounts in EOs extracted by HD with one exception, the EO from *O. majorana*, which surprisingly displayed 32% in the EO extracted by SD and was absent In the EO extracted by HD. However, interaction between the richness of the compounds can occur during the extraction process, making difficult the decision of which method employ.

## Conclusions

All the tested EOs from Lamiaceae and Asteraceae, except two, displayed good anti-trichomonal activity, showing the highest activity EOs from *L. luisieri, S. officinalis, S. hibrida, S. sclarea, T. vulgaris, T. zygis, O. majorana*, and *S. montana*.

Among the tested compounds in this study, six were selected as good candidates as AT drugs based on the absence of toxicity in cell cultures and the anti-trichomonad activity *in vitro*: linalyl acetate, thymol, 4-terpinenol, γ-terpinene, p-cymene, and D-fenchone. Three of them also showed a good correlation between the activity of the EOs and their abundance in the EO's composition: thymol, γ-terpinene, and p-cymene. Considering all the analysis conducted, their abundance in the selected plants EOs, the cytotoxic analysis and the AT activity, we propose these compounds as good anti-trichomonads candidates. Their activity could be increased with chemical modifications. Other compounds with high AT activity were α and β-pinenes, but their toxicity should be further studied.

However, although in most cases, the majority compounds of the EOs account for their activity, in some cases the antitrichomonal activity of the compounds is lower than that of the EOs, which could be due to their complex composition, where synergistic or antagonistic relationships occur between the components. The combination of various products could be of interest.

## Data availability statement

The original contributions presented in the study are included in the article/Supplementary material, further inquiries can be directed to the corresponding author/s.

## Author contributions

Conceptualization: AG-C, RM-D, MB, and MG-M. Data curation, supervision, writing, reviewing, and editing: AG-C, MB, and MG-M. Formal analysis: ID-C, MB, AG-C, and MG-M. Funding acquisition: AG-C, RM-D, and MG-M. Investigation: ID-C, IA-C, SA, and JN-R. Methodology: ID-C, IA-C, SA, JN-R, AG-C, MB, and MG-M. Resources: AG-C, JN-R, RM-D, MB, and MG-M. MG-M. Writing—original draft: ID-C and MG-M. All authors contributed to the article and approved the submitted version.

## Funding

This work has been partially financed by Grants PR108/20-08 (Santander-UCM), PID2020-114207RB-I00 (Spanish Ministry of Science and innovation), ERASMUS+ European Hub on New Challenges in the Field of Essential Oils (EOHUB) and PID2019-106222RB-C31/SRA (State Research Agency, 10.13039/501100011033).

## Conflict of interest

The authors declare that the research was conducted in the absence of any commercial or financial relationships that could be construed as a potential conflict of interest.

## Publisher's note

All claims expressed in this article are solely those of the authors and do not necessarily represent those of their affiliated organizations, or those of the publisher, the editors and the reviewers. Any product that may be evaluated in this article, or claim that may be made by its manufacturer, is not guaranteed or endorsed by the publisher.
