# Supplementary figures and images for: Anti-Trichomonas gallinae activity of essential oils and main compounds from Lamiaceae and Asteraceae plants
Source: Front Vet Sci. 2022 Sep 9;9:981763. doi: 10.3389/fvets.2022.981763 (PMC9500544; doi:10.3389/fvets.2022.981763)

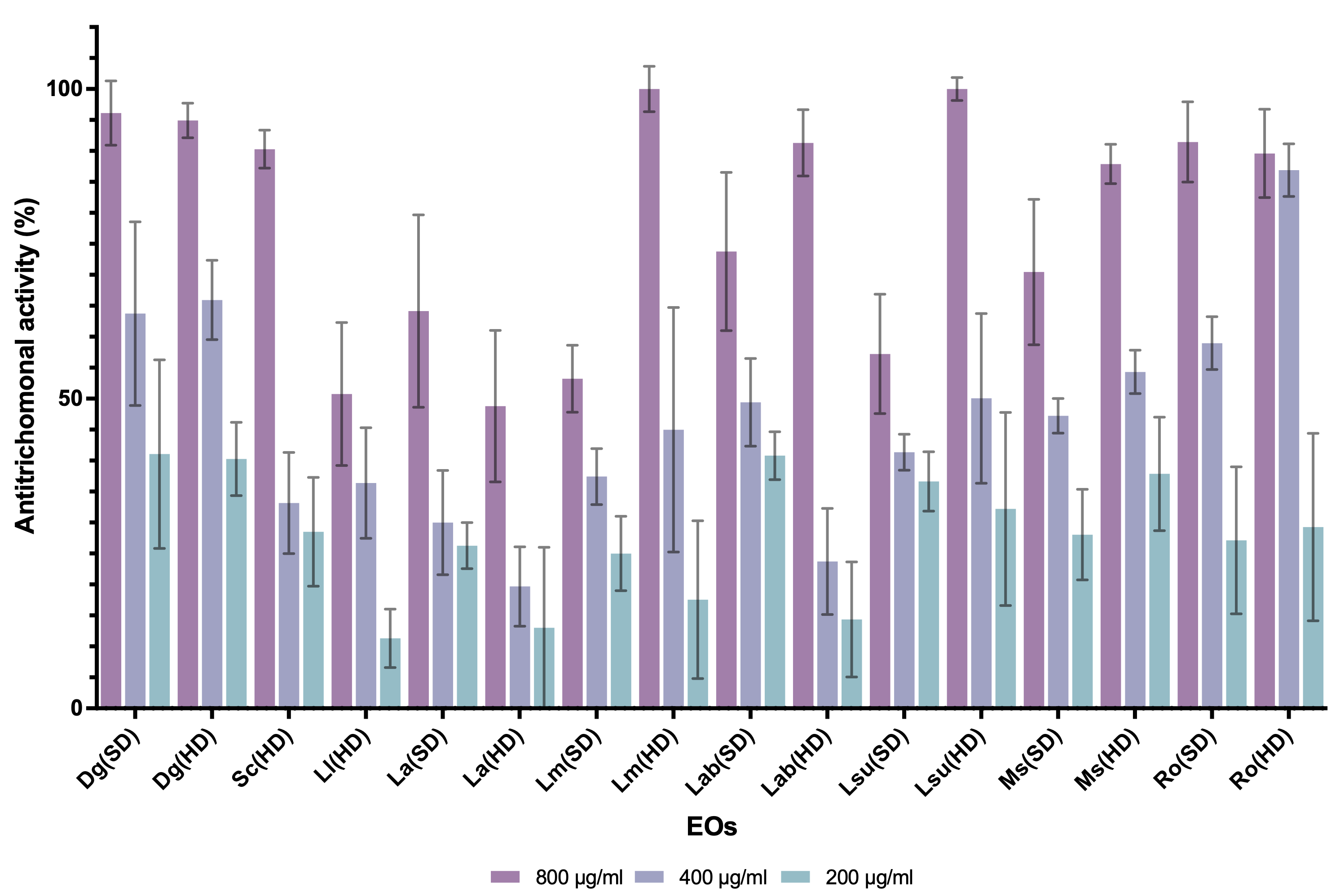

Supplement: Supplementary file 1 [file Image_1.TIFF]

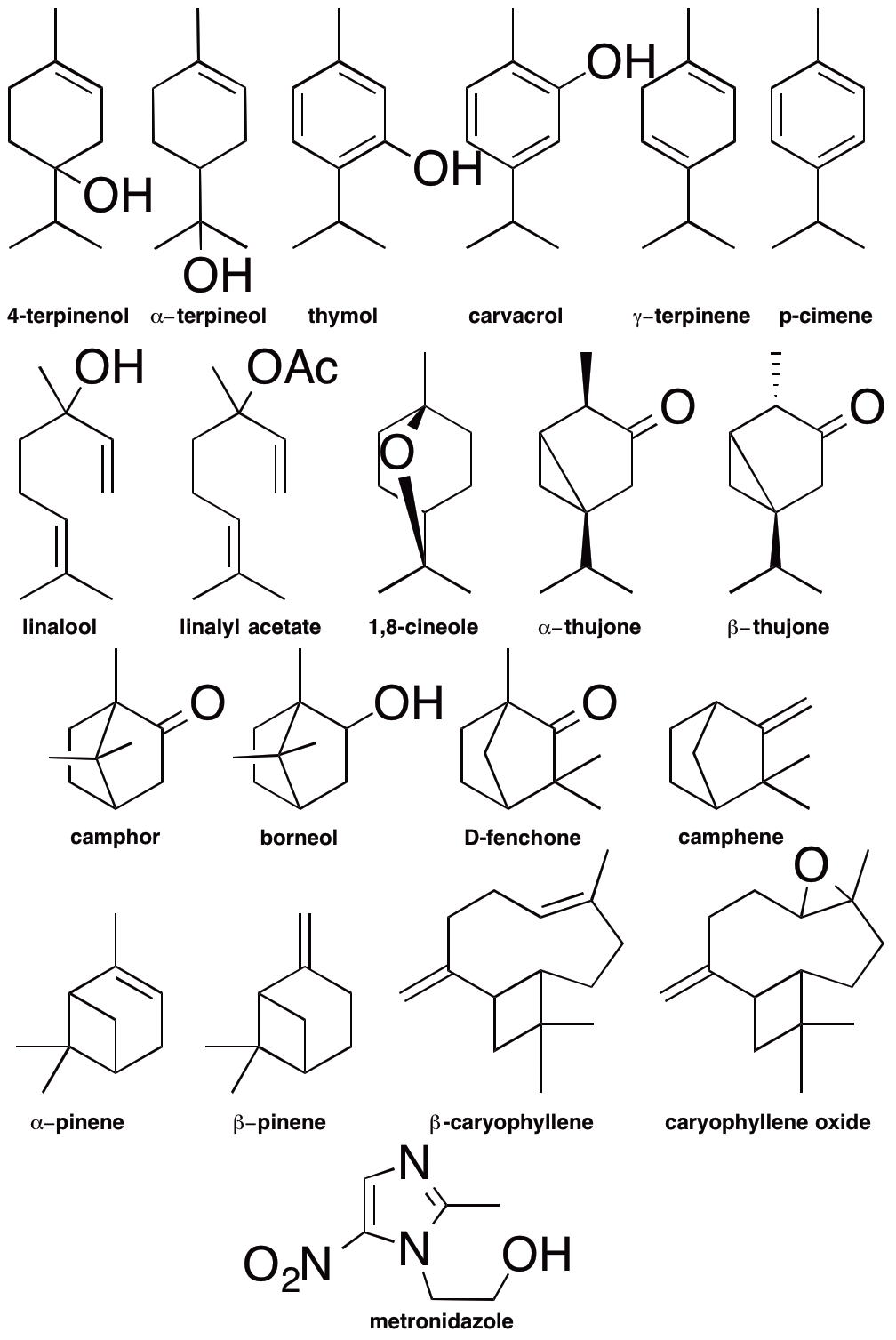

Supplement: Supplementary file 2 [file Image_2.TIF]
