# Supplementary material for: Anti-Trichomonas gallinae activity of essential oils and main compounds from Lamiaceae and Asteraceae plants
Source: Front Vet Sci. 2022 Sep 9;9:981763. doi: 10.3389/fvets.2022.981763 (PMC9500544; doi:10.3389/fvets.2022.981763)
Supplement: Supplementary file 3 [file Table_1.DOCX]

Table S1. Selected plant species, geographic location, coordinates and voucher numbers.

| **Family** | **Genus** | **Species** | **Location and coordinates** | **Voucher number** | **Extraction method** |
| --- | --- | --- | --- | --- | --- |
| Asteraceae | *Santolina* | *Santolina*  *chamaecyparissus* | Ejea de los Caballeros,  (42.13460057158429, -1.209717966420778) | JACA-R 310360 | HD |
|  | *Ditrichia* | *Ditrichia graveolens* | Puertollano, Spain (38.570457845640234, -4.427113119001229) | JACA-R310361 | SD |
|  |  | *Ditrichia graveolens* | Puertollano, Spain (38.570457845640234, -4.427113119001229) | JACA-R310361 | HD |
| Lamiaceae | *Lavandula* | *Lavandula lannata* | Ejea de los Caballeros,  Spain (42.13460057158429, -1.209717966420778) | JACA-R308581 | HD |
|  |  | *Lavandula luisieri* 1 | Aguaron, Spain (41.327393594227416, -1.313038546000645) | JACA-R310362 | SD |
|  |  | *Lavandula luisieri* 1 | Aguaron, Spain (41.327393594227416, -1.313038546000645) | JACA-R310362 | HD |
|  |  | *Lavandula luisieri* 2 | Aguaron, Spain (41.327393594227416, -1.313038546000645) | JACA-R310362 | SD |
|  |  | *Lavandula luisieri* 2 | Aguaron, Spain (41.327393594227416, -1.313038546000645) | JACA-R310362 | HD |
|  |  | *Lavandula angustifolia* | Ejea de los Caballeros,  Spain (42.13460057158429, -1.209717966420778) | JACA-R310363 | SD |
|  |  | *Lavandula angustifolia* | Ejea de los Caballeros,  Spain (42.13460057158429, -1.209717966420778) | JACA-R310363 | HD |
|  |  | *Lavandula x intermedia*  *“Abrial”** | Ejea de los Caballeros,  Spain (42.13460057158429, -1.209717966420778) | JACA-R310364 | SD |
|  |  | *Lavandula x intermedia*  *“Abrial”** | Ejea de los Caballeros,  Spain (42.13460057158429, -1.209717966420778) | JACA-R310364 | HD |
|  |  | *Lavandula x intermedia*  *“Super”** | Ejea de los Caballeros,  Spain (42.13460057158429, -1.209717966420778) | JACA-R310365 | SD |
|  |  | *Lavandula x intermedia*  *“Super”** | Ejea de los Caballeros,  Spain (42.13460057158429, -1.209717966420778) | JACA-R310365 | HD |
|  |  | *Lavandula mallete* | Ejea de los Caballeros,  Spain (42.13460057158429, -1.209717966420778) | JACA-R310366 | SD |
|  |  | *Lavandula mallete* | Ejea de los Caballeros,  Spain (42.13460057158429, -1.209717966420778) | JACA-R310366 | HD |
|  | *Origanum* | *Origanum virens* | Bernues, Spain (42.47712991851521, -0.5741776808332965) | JACA-R310367 | SD |
|  |  | *Origanum virens* | Bernues, Spain (42.47712991851521, -0.5741776808332965) | JACA-R310367 | HD |
|  |  | *Origanum majorana* | Ejea de los Caballeros,  Spain (42.13460057158429, -1.209717966420778) | JACA-R308580 | SD |
|  |  | *Origanum majorana* | Ejea de los Caballeros,  Spain (42.13460057158429, -1.209717966420778) | JACA-R308580 | HD |
|  | *Rosmarinus* | *Rosmarinus officinalis* | Jaraba (41.233626050102, -1.8987373367603422) | JACA-R310368 | SD |
|  |  | *Rosmarinus officinalis* | Ejea de los Caballeros,  Spain (42.13460057158429, -1.209717966420778) | JACA-R310369 | HD |
|  | *Satureja* | *Satureja montana* | Bernues, Spain (42.47712991851521, - 0.5741776808332965) | JACA-R310370 | SD |
|  |  | *Satureja montana* | Ejea de los Caballeros,  Spain (42.13460057158429, -1.209717966420778) | JACA-R310371 | HD |
|  | *Mentha* | *Mentha suaveolens* | Ejea de los Caballeros,  Spain (42.13460057158429, -1.209717966420778) | JACA- R235982 | SD |
|  |  | *Mentha suaveolens* | Ejea de los Caballeros,  Spain (42.13460057158429, -1.209717966420778) | JACA-R235982 | HD |
|  | *Salvia* | *Salvia officinalis* | Ejea de los Caballeros,  Spain (42.13460057158429, -1.209717966420778) | JACA-R308575 | SD |
|  |  | *Salvia officinalis* | Ejea de los Caballeros,  Spain (42.13460057158429, -1.209717966420778) | JACA-R308575 | HD |
|  |  | *Salvia hibrida* | Ejea de los Caballeros,  Spain (42.13460057158429, -1.209717966420778) | JACA-R308577 | SD |
|  |  | *Salvia hibrida* | Ejea de los Caballeros,  Spain (42.13460057158429, -1.209717966420778) | JACA-R308577 | HD |
|  |  | *Salvia sclarea* | Ejea de los Caballeros,  Spain (42.13460057158429, -1.209717966420778) | JACA-R308573 | SD |
|  |  | *Salvia sclarea* | Ejea de los Caballeros,  Spain (42.13460057158429, -1.209717966420778) | JACA-R308573 | HD |
|  | *Thymus* | *Thymus vulgaris* | Jaraba (41.233626050102, -1.8987373367603422) | JACA-R310372 | SD |
|  |  | *Thymus vulgaris* | Ejea de los Caballeros,  Spain (42.13460057158429, -1.209717966420778) | JACA-R310373 | HD |
|  |  | *Thymus zygis* | Ejea de los Caballeros,  Spain (42.13460057158429, -1.209717966420778) | JACA-R310374 | SD |
|  |  | *Thymus zygis* | Ejea de los Caballeros,  Spain (42.13460057158429, -1.209717966420778) | JACA-R310374 | HD |

*Sterile clones multiplied by cuts.
